# Cascade Screening for Familial Hypercholesterolemia (FH)

**DOI:** 10.1371/currents.RRN1238

**Published:** 2011-05-23

**Authors:** Renée M. Ned, Eric. J.G. Sijbrands

**Affiliations:** ^*^Health Scientist, Office of Public Health Genomics, Centers for Disease Control and Prevention, Atlanta, GA and ^†^Professor of Medicine, Erasmus MC, Rotterdam, The Netherlands

## Abstract

Familial hypercholesterolemia (FH) is an autosomal dominant disorder characterized by abnormally high concentrations of low-density lipoprotein (LDL) cholesterol in the blood, which predisposes affected persons to premature coronary heart disease (CHD) and death. FH is one of the most common inherited disorders and the most common one known to cause premature CHD in people of European descent. The vast majority of people with FH have inherited a single mutation from one parent in either the LDL receptor (LDLR), apolipoprotein B (APOB), or proprotein convertase subtilisin/kexin type 9 (PCSK9) genes. Despite their greatly elevated risk of coronary heart disease, most individuals with FH remain undiagnosed, untreated, or inadequately treated.

Cascade screening is a mechanism for identifying people at risk for a genetic condition by a process of systematic family tracing. The National Institute for Health and Clinical Excellence in the United Kingdom recommends cascade screening of close biological relatives of people with a clinical diagnosis of FH in order to effectively identify additional FH patients. The ultimate goal of this testing is to reduce morbidity and mortality from heart disease in persons with FH through early diagnosis and effective disease management. The goal of this article is to outline the available evidence on the clinical validity and utility of cascade screening for FH, while emphasizing the availability, usefulness, and recommendation for including DNA testing (if the disease-causing mutation has been identified).

**Clinical Scenario**

A patient has been diagnosed with familial hypercholesterolemia (FH), Use cascade screening to identify biological relatives of the patient who are also affected with the disorder.    

**Test Description**

Identifying and contacting biological relatives of a person diagnosed with FH (the index case) and then systematically testing these relatives (first-, second-, third-, etc. degree) using a combination of serum LDL cholesterol concentration measurements and a variety of mutation detection or screening assays for mutations in the *LDLR*, *APOB*, or *PCSK9* genes.  

**Public Health Importance**

FH is one of the most common inherited disorders, with an estimated worldwide prevalence of 1 in 500 (0.2%), though the frequency is considerably higher in some populations because of a founder effect [1]. This estimate corresponds to approximately 13 million people worldwide and ~600,000 in the United States who have FH. The overwhelming majority of affected persons are heterozygotes (those who have inherited one disease-causing mutation). A smaller number of patients (~1 in 200,000) are compound heterozygotes (who have inherited one copy each of two different mutations), while persons with homozygous FH (who have inherited two identical disease-causing mutations) are extremely rare (~1 in 1 million).  

The elevated serum cholesterol levels associated with FH lead to a greatly elevated risk for coronary heart disease (CHD) and death [2]. In fact, for those with heterozygous FH, the cumulative risk for CHD is greater than 50% in men by the age of 50 and at least 30% in women by the age of 60 [3] [4]. Persons with homozygous FH manifest an even more severe form of the disorder. There is variation in the onset and severity of atherosclerotic disease in persons with FH, since environmental, metabolic, and genetic factors influence the clinical phenotype [2] [5] [6].  

Overall, estimates are that fewer than 25% of persons with FH are diagnosed; and the majority remain untreated or improperly treated [7] [8] [9], though there are no recent estimates available for the United States [10]. (Persons may be treated for high cholesterol without knowledge that they have FH. False-negative diagnoses can result in inadequate treatment, while false-positive diagnoses may result in overtreatment.) 

Because of the high prevalence of FH among family members (50% of first-degree relatives of heterozygotes are affected), cascade screening has been shown to be a cost-effective method of identifying people with FH [3] [11] [12]. Early detection and treatment with statins have been shown to reduce morbidity and mortality among those with heterozygous FH [3] [4] [11]. 

Despite an international effort to improve the identification and management of patients with FH [7] [9], few countries have established large-scale programs to systematically determine the FH status of relatives of these patients [13] [14] [15] [16] [17] [18] [19] [20] [21].    

**Published Reviews, Recommendations and Guidelines**

**Systematic evidence reviews**

A systematic evidence review [3] was conducted during the formulation of guidelines from the National Institute for Health and Clinical Excellence (NICE) in the United Kingdom (see next subsection).   

**Recommendations by independent group**

The NICE guidelines on the identification and management of FH recommend cascade screening using a combination of genetic testing and LDL cholesterol concentration measurement "is recommended to identify affected relatives of those index individuals with a clinical diagnosis of FH. This should include at least the first- and second- and, when possible, third-degree biological relatives” [3] [22].  

Currently, there are no formal clinical guidelines or recommendations in the United States regarding cascade screening for FH.    

**Guidelines by professional groups**

None.   

**Evidence Overview**

***Analytic Validity*: **Test accuracy and reliability in measuring the genomic markers of interest— mutations in the *LDLR*, *APOB*, or *PCSK9* genes (analytic sensitivity and specificity). 

- Mutations known to cause FH have been found in the LDL receptor (*LDLR*), apolipoprotein B (*APOB*), and proprotein convertase subtilisin/kexin type 9 (*PCSK9)* genes. Several methods are currently used to identify sequence changes in these genes [3] [23] [24]; and additional technologies are under development.
- The analytic sensitivity and specificity of each DNA test for FH will depend on the particular mutations being assayed and on the population(s) tested. (Often, labs will initially test only for those mutations that are most prevalent in a particular population.)
- Because of the number of common polymorphisms that exist and because some scanning methods are not very efficient in detecting large-scale genetic rearrangements, many diagnostic labs are switching to direct sequencing strategies to improve the sensitivity of their mutation detection efforts [23].

***Clinical Validity*: **Test accuracy and reliability in identifying relatives of patients with FH (predictive value).

- More than 1,000 mutations in the *LDLR* gene have been identified in patients with FH [24]; and DNA testing on this gene alone has been found to identify 70-80% of people with a definitive clinical diagnosis of FH and 20-30% of those whose diagnosis is less certain [3] [24]. Researchers have estimated that mutations in the *APOB* and *PCSK9* genes account for only about 5.5% and 1.5% of FH cases, respectively [24].
- Current DNA testing for FH is not 100% sensitive because the disorder may be caused by a mutation that is not assessed. Therefore, *not* finding a mutation does not necessarily exclude a diagnosis of FH [3]. Research has shown that ~15% of people with FH do not have a mutation in their *LDLR*, *APOB*, or *PCSK9* genes (though reliable estimates range from 12% to 48%) [24].
- Conversely, though mutation screening for FH is highly specific, it may produce false positives because some sequence changes that are detected can be non-pathogenic [23] [24] [25]. However, a false positive mutation will likely become evident during cascade screening, since it should fail to consistently associate with the FH phenotype.
- Since FH is a disease inherited in an autosomal dominant fashion, the identification of the true mutation responsible for the disease in a family allows for the definitive diagnosis of biological relatives who have the mutation [3] (i.e., 100% positive predictive value).
- If a familial FH mutation is not identified or if genetic testing is not available, relatives of an FH patient can be diagnosed with the disorder on the basis of sex- and age-specific LDL cholesterol thresholds (as recommended by NICE) [3] [22]. However, because of an overlap in LDL cholesterol levels between people with FH and those without, there is still diagnostic uncertainty in over 15% of individuals [3] [26].  Relatives can also be diagnosed on the basis of specific clinical characteristics, such as the presence of xanthomas (though these are not present in all persons with FH).
- Published studies on the clinical validity of diagnosing relatives using DNA-based criteria compared to clinical criteria have shown that a clinical diagnosis is less sensitive and leads to under-diagnosis [15] [27] [28] [29], raising the possibility that relatives diagnosed by clinical criteria may, in fact, have another form of dyslipidemia (i.e., they may have been misdiagnosed).

***Clinical Utility*: **Net benefit of cascade screening in improving health outcomes.

- Data from the U.K. has shown that cascade screening reduces the average age at which FH patients are diagnosed [3].
- Cascade screening has also resulted in increased percentages of people with FH on statins and has, subsequently, resulted in decreased lipid levels in these people [3] [27] [30].
- Statin use has been shown to reduce both total cholesterol and LDL cholesterol in adults with FH; and early detection and treatment with statins have been shown to reduce morbidity and mortality among those with heterozygous FH [3] [4] [11]. In one of the few long-term cohort studies of FH patients, there was an ~80% reduction in the risk of first onset of CHD among those treated with statins compared to untreated patients [31]. This study mimicked a placebo-controlled primary prevention trial and is the highest quality evidence of the effect of statin treatment in FH patients. Results from another long-term prospective study examined mortality in heterozygous FH patients aged 20-79 years old and showed an overall 37% reduction in deaths from CHD since statins became widely available. (The reduction in coronary mortality was greater for primary CHD prevention than for secondary CHD prevention— 48% versus 25% reduction) [4]. Other researchers used the concept of population attributable fraction to estimate the proportion of the 5-year risk for CHD mortality that could potentially be prevented among first-degree relatives of FH patients by treating the hypercholesterolemia among the affected relatives. This proportion was 44% among male, and 57% among female, first-degree relatives aged 20-79 years [32]. Remarkably, this analysis estimated that 96-98% of the deaths due to CHD among those under the age of 40 could be prevented with cholesterol reduction [32].
- Statin treatment has been shown to decrease total and LDL cholesterol concentrations in children with FH [3] [33] [34] [35] [36] [37] [38]. However, there is no evidence of the long-term health benefits of statin treatment in children with FH (such as delayed onset of, or reduced risk for, CHD), nor evidence of any benefits compared to the detection and treatment of the disorder in adulthood [35] [39]. Nevertheless, there are studies that support cholesterol lowering in children based on surrogate markers of cardiovascular disease [such as carotid intima-media thickness (IMT)] [40] [41] [42] [43], some of which note a significant deviation in carotid IMT from the age of 12 years in children with FH (compared to unaffected siblings) [42].  Increased carotid IMT is reversible by statins, though we lack information regarding when such changes become irreversible. 
- Because cascade screening may identify fewer than 50% of people with FH (at least as estimated for the U.K. population) [3], research is needed to compare the utility of other strategies for identifying those with FH.
- There are no published data on the clinical utility of cascade screening that includes DNA testing compared to cascade screening without genetic analysis.
- Hard data are also lacking regarding whether FH identification by cascade screening (and subsequent treatment of FH patients) is more effective in improving health outcomes of these patients compared to other identification methods (e.g., general physician note searching, use of secondary care registries, or population screening).
- The following issues may also affect the ability of cascade screening to reduce CHD and CHD-related deaths among relatives of FH patients:

- **The identification and diagnosis of index cases.** It is unclear whether a screening program should be implemented to find FH index cases and, if so, what type of program would be best (e.g., population-based or opportunistic screening of adults; universal or targeted lipid screening of children) [11] [12] [35] [44] [45] [46] [47] [48]. In addition, three groups have developed diagnostic criteria for FH. These criteria differ in their need for (and the sufficiency of) DNA testing and in their diagnostic effectiveness [1] [3].
- **The possibility of variation in the severity of FH phenotypes according to the type of mutation (the genotype-phenotype relationship), particularly for *LDLR*.** For example, LDL receptor-null mutations have been associated with higher blood LDL cholesterol levels compared with LDL receptor-defective mutations [2] [43] [49] [50] [51]. However, family-based and population-based studies have largely found that statin treatment efficacy or the risks of CHD or CHD-related mortality are not affected by *LDLR *mutation type [49] [52] [53]. Of note, the only subgroup analysis performed in a randomized, double-blinded trial showed that *LDLR* mutation type altered the LDL-lowering effect of the investigated statin, but this trend was not statistically significant [50].
- **Uncertainty surrounding when and how to pharmacologically treat lipid disorders in children.** As noted previously, there is no evidence of the long-term health benefits or safety of statin treatment in children, as the longest follow-up period in studies was 2 years [33] [34] [36] [37] [38]. Therefore, even if a child with FH is identified through cascade screening, the issue of when to start drug therapy is not straightforward. The U.S. Preventive Services Task Force (USPSTF) has not issued specific recommendations for the treatment of children with FH, although it did note that statins are effective for reducing total and LDL cholesterol levels in these children [35] [44]. The American Academy of Pediatrics recommends that in children and adolescents with heterozygous FH, initial intervention “is focused on changing the diet. However, if this approach does not lower LDL to an acceptable concentration, these children may be candidates for pharmacologic intervention” [46]. The American Heart Association is more aggressive, recommending the consideration of lipid-lowering therapy for children aged ≥10 years (and after the onset of menses for girls) whose LDL levels are “severely elevated” and that the age or LDL cutpoint at which such therapy is initiated may be even lower for children with additional cardiovascular risk factors [48] [54]. The recommendation from NICE is similar: “lipid-modifying drug therapy for a child or young person with (heterozygous) FH should usually be considered by the age of 10 years”, taking into account the presence of other cardiovascular risk factors and the family history of CHD [3] [22].

- It is important to note, however, that lifestyle interventions are also an important component of the medical management of FH for both children and adults
- [22]
- , since environmental and metabolic factors can influence the FH phenotype
- [5]
- [6]
- .  FH patients (including children) are encouraged to get adequate physical activity, eat a healthy diet, and to refrain from smoking.  Of note, however, a recent Cochrane review of randomized controlled trials found that currently available data are insufficient to reach any conclusions regarding the efficacy of different dietary interventions for FH patients, including a lack of data on the usefulness of dietary modification over and above lipid-lowering drug therapy
- [55]
- .

      **The best means by which to contact relatives of an FH index case.** This subject is currently under debate [56] [57]. 

In summary, the clinical validity and utility of cascade screening for FH is dependent on a number of factors, including the criterion used to diagnose the disorder in the index case, the use of DNA testing in the index case and in relatives, and the nature of the benefit and possible harms of identifying and pharmacologically treating the disorder in childhood.  Nevertheless, cascade screening is a straightforward and highly effective way to identify persons who have FH.   

**Links**

- Online Mendelian Inheritance in Man (OMIM) entry on Hypercholesterolemia, Autosomal Dominant: http://www. http://www.ncbi.nlm.nih.gov/omim/143890.
- NICE guidelines: http://www.nice.org.uk/guidance/CG71.
- Make Early Diagnosis to Prevent Early Death (MEDPED): www.medped.org; [58].
- CDC podcast on cascade screening for FH: http://www.cdc.gov/genomics/resources/video/RNed/index.htm.

Last updated: May 23, 2011    

**Acknowledgments**

We would like to thank Shelley Reyes, Nicole F. Dowling, and Cecelia Bellcross in the Office of Public Health Genomics (OPHG) at CDC for comments and guidance.    

**Funding information**

This work was supported by the Office of Public Health Genomics (OPHG), Office of Surveillance, Epidemiology, and Laboratory Services (OSELS), Centers for Disease Control and Prevention (CDC).  

**Competing interests**

The authors declare that no competing interests exist.  

**Disclaimers**

The findings and conclusions in this report are those of the authors and do not necessarily represent the views of the Centers for Disease Control and Prevention (CDC).  

The CDC does not offer medical advice to individuals. If you have specific concerns about your health or genetic testing, we suggest that you discuss them with your health care provider. 
